# A new species of *Aleuromarginatus* Corbett, 1935 with a key and checklist of Chinese species (Hemiptera, Aleyrodidae)

**DOI:** 10.3897/zookeys.682.11767

**Published:** 2017-07-05

**Authors:** Ji-Rui Wang, Zhi-Hong Xu, Yu-Zhou Du

**Affiliations:** 1 School of Agricultural & Food Science, Zhejiang Agriculture & Forestry University, Linan, Zhejiang 311300, China; 2 School of Horticulture and Plant Protection & Institute of Applied Entomology,; 3 Yangzhou University, Yangzhou, Jiangsu 225009, China

**Keywords:** Aleyrodidae, *Aleuromarginatus*, China, new taxa, taxonomy

## Abstract

A new whitefly species, *Aleuromarginatus dielsianae* Wang & Xu, **sp. n.** collected from *Millettia dielsiana* Harms (Rosales: Fabaceae) in Jiangshan (28°40'N, 118°40'E, 512 m) and Xinchang (29°22'N, 120°46'E, 308 m), Zhejiang, China, is described and illustrated. This new species is characterized by the dark brown lateral margin area and a pair of longitudinal furrows extending from the cephalothorax to the vasiform orifice. The submargin has an elongate-oval fold at the base of each marginal tooth and with 3-4 rows of irregular shaped papillae, nine pairs submedian setae and 13 pairs submarginal setae. Thoracic and caudal tracheal folds and pores discernible. An identification key and checklist of species of *Aleuromarginatus* known from China are provided.

## Introduction

The genus *Aleuromarginatus* (Hemiptera: Aleyrodidae) was established by Corbett (1935) with *A. tephrosiae* Corbett as the type species by monotypy. *Aleuromarginatus* is very distinct; based on the special characteristics of the puparium it unlikely to be confused with other whitefly genera. It is an Old World genus, recorded from the Afrotropical, Palearctic, Oriental, and Australasian regions. Only 14 species of this genus have been described, almost all of them are known only from leguminous plants (Fabaceae) (Bink-Moenen 1983; Cohic 1968, 1969; Corbett 1935a; David 1976, 1988; David and Subramaniam 1976; Jesudasan and David 1991; Ko et al. 1995; Martin 1985, 1999; Martin and Mound 2007; Mound and Halsey 1978; Takahashi 1955).

This genus was unknown from East Asian region until Ko et al. (1995) described *Aleuromarginatus shihmensensis* Ko on *Millettia seiculata* from Taiwan. In addition, *A. thirumurthiensis* David is known to occur in Taiwan (Chiun-Cheng Ko personal collection) and Wang et al. (2016) recorded *A. corbettiaformis* Martin from Hainan Island of China. In this paper, the fourth species of *Aleuromarginatus* from China is described.

## Material and methods

Puparia of the new species were collected from *Millettia dielsiana* Harms (Rosales: Fabaceae) in Shuangxikou village, 28°40'N, 118°40'E, 512 m, Jiangshan and Jingling town, 29°22'N, 120°46'E, 308 m, Xinchang, Zhejiang, China. The puparia were mounted following the method suggested by Dubey and David (2012). The terminology for morphological structures follows Bink-Moenen (1983), Martin (1985) and Gill (1990). The habitus images were taken using the digital camera Canon IXUS 105 and LEICA M125 stereo-microscope (Leica, Wetzlar, Germany) attached with a LEICA DFC290 (Leica, Wetzlar, Germany). Puparial measurements and microphotographs were taken using a Zeiss (Carl Zeiss, Gottingen, Germany) from ZAFU. The scanning electron microscope images were taken by Hitachi TM-1000 Scanning Electron Microscope (Hitachi, Japan) from Center of Electron Microscopy, Zhejiang University (Life Sciences Division). Adobe Photoshop software was used to make small adjustments and to assemble the plates. The holotype is deposited in the Insect Collection of Zhejiang Agriculture & Forestry University, Lin’an, China (ZAFU).

## Taxonomy

### Aleuromarginatus

Taxon classificationAnimaliaHemipteraAleyrodidae

Corbett, 1935

Aleuromarginatus Corbett 1935: 246. Type species. Aleuromarginatus tephrosiae, by monotypy.

#### Diagnosis.

Puparia elongate to broadly oval, often slightly indented anteriorly and posteriorly and/ or at thoracic tracheal openings at margin (Martin 1999); margin with two rows of teeth and surrounded by a waxy palisade and fringe of wax-hairs; submarginal area not separated from dorsal disc. Dorsal with a subdorsal and submedian row of short setae including the cephalic, first and eighth abdominal setae; vasiform orifice cordate, operculum filling about half of orifice, lingula knobbed, exposed; caudal furrow faint (Jesudasan and David 1991; Ko et al. 1995). This genus resembles *Aleurotrachelus* in the two- teethed margin, and resembles *Crenidorsum* with the submedial furrow or papillae, but can be distinguished by the characters of the vasiform orifice and the absence of spine-like setae on the medial region of the dorsum.

### Aleuromarginatus dielsianae

Taxon classificationAnimaliaHemipteraAleyrodidae

Wang & Xu sp. n.

http://zoobank.org/4CDE85C3-0F93-4B91-AB73-871F7613AEB9

Figures 1–2 , 3–4 , 5–10 , 11–13 , 14–16

#### Type locality.

China, Zhejiang, Jiangshan, Shuangxikou village, 28°40'N, 118°40'E, 512 m, on *Millettia dielsiana* Harms, 8. viii. 2016, leg. JR Wang.

#### Type material.

Holotype. China, Zhejiang, Jiangshan, Shuangxikou village, 28°40'N, 118°40'E, 512 m, 1 puparium on slide, on *Millettia dielsiana* Harms, 8. viii. 2016, leg. JR Wang, deposited in Insect Collection of Zhejiang Agriculture & Forestry University (ZAFU), Lin’an, China.

Paratypes. 35 paratypes of which: 28 are puparia on 20 slides, data same as holotype and 7 are puparia on 5 slides collected in Jingling town, 29°22'N, 120°46'E, 308 m, Xinchang, Zhejiang, China, on *Millettia dielsiana* Harms, 12. xi. 2016, leg. JR Wang, deposited in ZAFU. 68 dry puparia on *Millettia dielsiana* Harms leaves with above collection data available at ZAFU.

**Figures 1–2.**
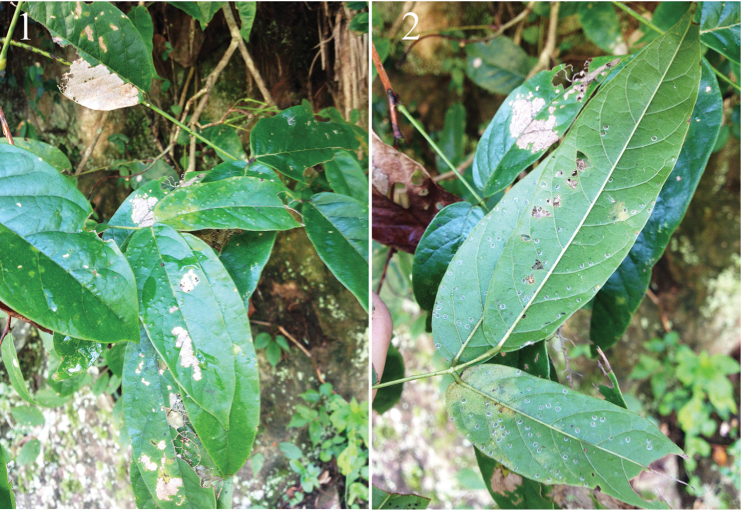
The host plant *Millettia dielsiana* Harms. **1** upper side of leaves infested by *Aleuromarginatus dielsianae* sp. n. **2** colony of *Aleuromarginatus dielsianae* sp. n. on the lower surface of leaves.

#### Diagnosis.

This species is characterized by the dark brown margin area (Figs 4, 11, 12), in life with a pair of longitudinal submedian lines (Fig. 4) and microscopically with a pair of longitudinal submedian furrows (Figs 5, 7, 8, 11) from cephalothorax to the vasiform orifice. Submargin with an elongate-oval fold at the base of each marginal tooth and with 3-4 rows of irregularly shape papillae (Figs 9, 12). Nine pairs submedian setae (Fig. 14), minute, blunt - one pair of cephalic setae (cs), two pairs of thoracic setae (ts), six pairs of abdominal segment I and III-VI, VIII (as1, 3-6, 8); 13 pairs submarginal setae (sms) (Fig. 14) - three cephalic pairs, five thoracic pairs, one abdominal pair, and four posterior pairs. Vasiform orifice cordate (Figs 10, 13, 16); operculum broadly trapezoidal, covering nearly half the orifice; lingula exposed, setose, knobbed. Paired posterior marginal setae present while anterior marginal setae absent. Thoracic and caudal tracheal folds and pores discernible (Figs 6, 14).

**Figures 3–4.**
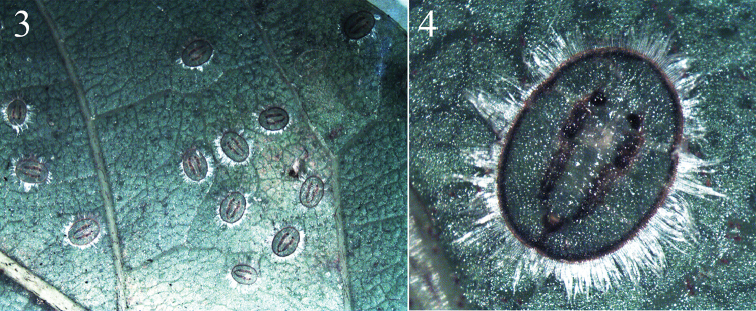
Images of pupaira of *Aleuromarginatus dielsianae* sp. n., on *Millettia dielsiana* leaves.

**Figures 5–10.**
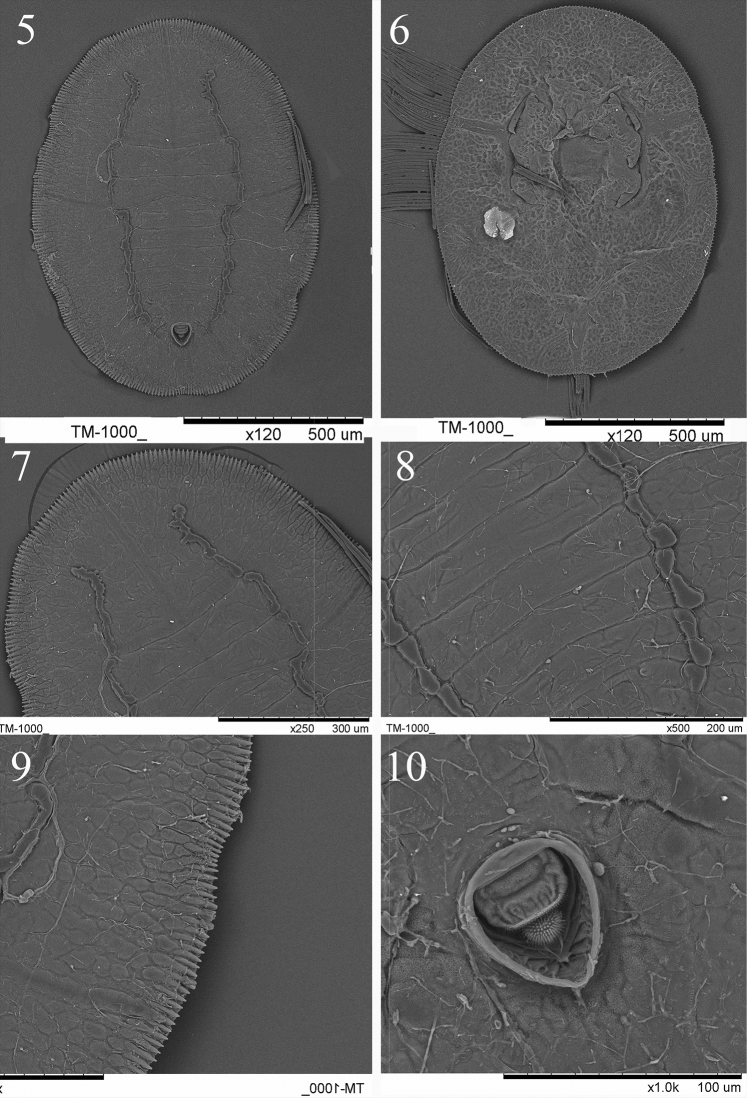
Scanning Electron Microscope photographs of *Aleuromarginatus dielsianae* sp. n. **5** puparium, dorsal view. **6** puparium, venter view **7** the longitudinal furrows on cephalothorax **8** the longitudinal furrows on abdomen and the abdomen segments **9** margin **10** vasiform orifice, operculum and lingula.

#### Description.

**Puparium.** Puparia have highly characteristic secretions in the form of a broad, laterally directed, white fringe on each side of the body, the fringe about 0.24-0.29 mm long; body light yellowish, transparent, margin dark brown; two longitudinal pigmented bands encompassing the submedian zone on either side of the body lines from cephalothorax to vasiform orifice; elliptical, 1.08-1.12 mm long, 0.75–0.82 µm wide, broadest at the abdominal segments I region. The presence of a colony can be easily ascertained by the dense bumps on the upper surface of the leaves caused by the puparia which embed themselves into the under surface of leaves, pushing the top surface of the leaf upward (Fig. 1).

Margin (Figs 9, 12, 15) strongly toothed, with a pore at the base of each 3–4 teeth, 13–15 crenulations in 0.1 mm. The thoracic and caudal tracheal areas slightly recessed and differentiated from margin. Paired posterior marginal setae (pms) present, about 35.1 µm long, anterior marginal setae absent.

**Figures 11–13.**
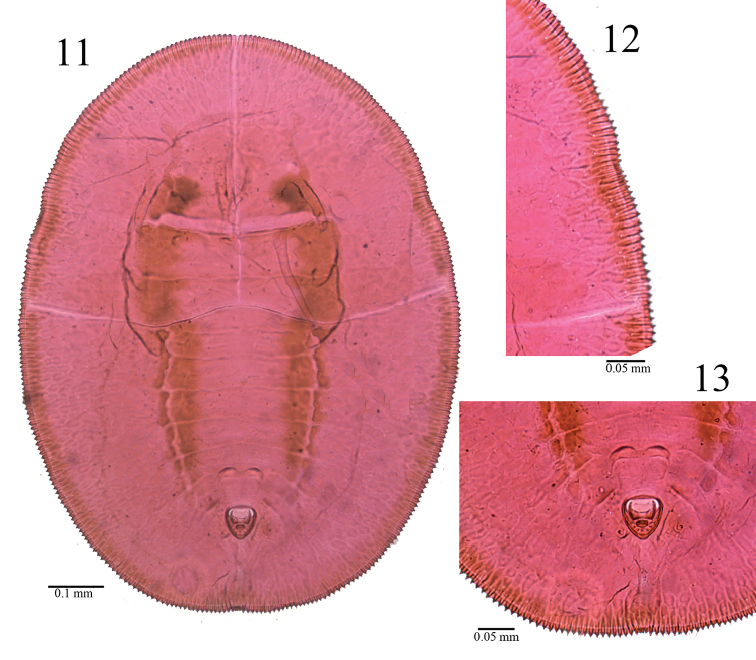
*Aleuromarginatus dielsianae* sp. n., slide mounted specimen. **11** puparium, dorsal view **12** margin **13** vasiform orifice, operculum and lingula.

*Dorsum* almost flat, without tubercle, sparsely scattered with pores. Submarginal area not clearly separated from dorsal disk. A pair of dark brown longitudinal furrows (Figs 4, 5, 7, 8, 11) extending from the cephalic region to the vasiform orifice, the longitudinal furrows consist of some short longitudinal furrows. Submargin with an elongate-oval fold at the base of each marginal tooth and with 3–4 rows of irregular shape papillae (Figs 9, 12). Nine pairs submedian setae, minute, blunt - one pair of cephalic setae (cs), two pairs of thoracic setae (ts2, 3) which are on the 2^nd^ and 3^rd^ thoracic segments; six pairs of abdominal setae, one pair on each segments I and III-VI, VIII (as 1, 3–6, 8). Thirteen pairs submarginal setae (sms) - 3 cephalic pairs, 5 thoracic pairs, 1 abdomen pair and 4 posterior pairs. The submedian setae and submarginal setae each arising from a small tubercle and are subequal in length, about 6.1–6.7 µm. Longitudinal and transverse molting sutures reaching the anterior and lateral margin, respectively. The transverse molting suture slightly protruding forming a transverse ridge (Fig. 7). Thorax and abdominal segment sutures well defined, midline of abdominal segments I-II each about 44.5 µm in length; abdominal segments III-IV each about 54.8 µm in length; abdominal segments V about 47.3 µm in length; abdominal segments VI about 35.6 µm in length; abdominal segments VII about 12.7 µm in length.

Vasiform *orifice* (Figs 10, 13, 16) cordate, longer than wide, 65.5–68.3 µm long, 60.2–62.3 µm wide; operculum broadly trapezoidal, covering nearly half the orifice, 29.5–34.8 µm long, 39.2–41.2 µm wide. Lingula exposed, setose, knobbed, 9.1–12.2 µm long, 13.6–16.4 µm wide, with a pair of apical setae, about 7.4 µm in length.

*Venter*. Thoracic and caudal tracheal folds and pores discernible (Fig. 6). Ventral abdominal setae placed on either side of anterior angles of vasiform orifice, finely pointed and 5.7–7.8 µm long, 53.1 µm apart. Antenna slender, long, extending slightly beyond the prothoracic spiracular furrow but not reaching base of mesothoracic leg.

**Figures 14–16.**
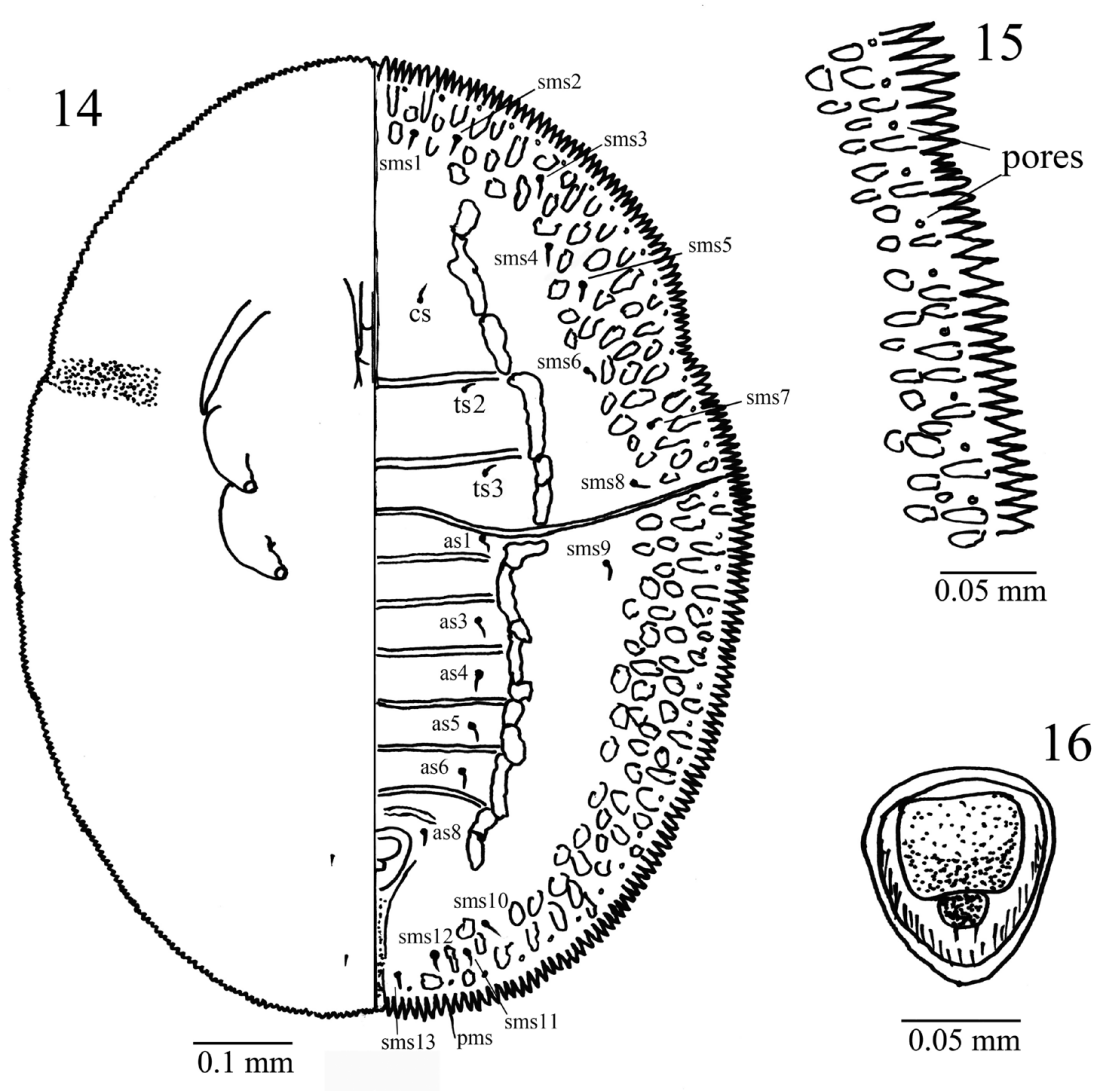
*Aleuromarginatus dielsianae* sp. n., holotype puparium, China (Zhejiang). **14**puparium, dorsal (right) and ventral (left) views **15** margin and minute pores **16** vasiform orifice.

#### Host plant.

*Millettia dielsiana* Harms (Figs 1, 2) (Rosales: Fabaceae).

#### Distribution.

China (Zhejiang).

#### Biology.

Specimens were found on the leaves in colonies from 20 - 60 individuals, distributed throughout the under surface of leaves (Fig. 2). No parasitoids were obtained from the puparia and no ants were observed attending the whiteflies.

#### Etymology.

The species name takes its name of host plant *Millettia dielsiana* Harms.

#### Remarks.

The new species resembles *A. millettiae* Cohic but differs in that the longitudinal furrows extend from the submendian region of the cephalic to the vasiform orifice while they are only present on the abdomen for *A. millettiae* and differs in the number and postion of the submarginal setae. The new species also resembles *A. kallarensis* David & Subramaniam but can be easily distinguished by the shape and the size of the puparia.

### Key to the puparia of Chinese species of *Aleuromarginatus*

(Characters are obtained from original descriptions)

**Table:** 

1	Puparia elongate-oval in shape	2
–	Puparia elliptical or roundish in shape	3
2	Dorsum cuticle brownish. Vasiform orifice cordate, anterior and posterior margin straight, lateral margins almost rounded, operculum roundly trapezoidal; lingula setose, knobbed, exposed but included. Pupal case ♀1.44–1.68mm long, 0.45–0.52mm wide; ♂ 1.04–1.28mm long, 0.38–0.4mm wide; on average 2.7–3.2 times as long as wide. Known only feeding on plant *Millettia reticulata*	***A. shihmensensis* Ko**
–	Dorsum cuticle pale, but many specimens with a brown median stripe. Vasiform orifice subcordate, anterior and posterior margin rounded, lateral margins almost straight, operculum trapezoidal with rounded lateral margins; lingula with large spinulose head, occupying most of the remaining area of the orifice. Pupal case ♀1.40–1.55mm long, 0.65–0.7mm wide; ♂ 1.10–1.2mm long, 0.47–0.5mm wide; on average 2.3 times as long as wide. Known only to feed on *Desmodium umbellatum*	***A. corbettiaformis* Martin**
3	Puparia elliptical, dorsum with a pair of the longitudinal furrows extending from the cephalus to the vasiform orifice region. Margin with numerous, long, pointed teeth, with a pore at the base of each set of 3–4 teeth, 13–15 crenulations in 0.1 mm; anterior marginal setae absent. Thoracic and caudal tracheal folds and pores discernible	***A. dielsianae* Wang & Xu, sp. n.**
–	Puparia oval to roundish, a row of papillae-like markings evident on subdorsum laterally from the posterior end of cephalic region to level of eighth abdominal segment. Margin strongly toothed with a pore at the base of each tooth, 24–27 crenulations in 0.1 mm; anterior marginal setae present. Thoracic and caudal tracheal folds and pores indiscernible	***A. thirumurthiensis* David**

### Checklist of Chinese species of *Aleuromarginatus*

**1. *Aleuromarginatus corbettiaformis* Martin, 1985**

Reported from China (Hainan Island) by Wang et al. (2016), voucher material in YZU from an unidentified Leguminosae plant.

**2. *Aleuromarginatus dielsianae* Wang & Xu, sp. n.**

Jiangshan and Xinchang, Zhejiang Province, China on *Millettia dielsiana*.

**3. *Aleuromarginatus shihmensensis* Ko, 1995**

Described from Taiwan by Ko (1995), holotype on *Millettia reticulata* in National Taiwan University (NTU).

**4. *Aleuromarginatus thirumurthiensis* David, 1988**

This species was first described on *Bauhinia racemosa* from India by David (1976) as “*Aleuromarginatus bauhiniae* David”. However, David (1988) transferred *Trialeurodes bauhinae* Corbett (Corbett 1935b) to the genus *Aleuromarginatus* thus making his 1976 species a junior homonym of *Aleuromarginatus bauhinae* (Corbett). Since the two species are clearly distinct species, a replacement name, *Aleuromarginatus thirumurthiensis*, was proposed by David (1988) for his 1976 species.Ken-Ching Chou collected this species from *Millettia reticulata* and *Bauhinia championii* in Taiwan in 1995, voucher material in NTU. (Chiun-Cheng Ko, pers. comm.).

## Supplementary Material

XML Treatment for Aleuromarginatus

XML Treatment for Aleuromarginatus dielsianae
